# Comparison of tablet versus liquid ethanol-free Levothyroxine in thyroidectomised patients

**DOI:** 10.1007/s12020-025-04375-9

**Published:** 2025-08-08

**Authors:** Laura Croce, Spyridon Chytiris, Marsida Teliti, Jacopo Bertini, Lidia Pizzuto, Marzia Dal Molin, Matteo Limiroli, Benedetto Calì, Flavia Magri, Mario Rotondi

**Affiliations:** 1 Department of Internal Medicine and Therapeutics, University of Pavia, Pavia, 27100 Italy; 2 Istituti Clinici Scientifici Maugeri IRCCS, Unit of Internal Medicine and Endocrinology, Laboratory for Endocrine Disruptors, Pavia, 27100 Italy; 3 Internal Medicine, Endocrinology and Metabolic Diseases Unit, IRCCS Ospedale Galeazzi – Sant’Ambrogio, Milan, 20157 Italy; 4 Istituti Clinici Scientifici Maugeri IRCCS, Department of General and Minimally Invasive Surgery, Pavia, 27100 Italy

**Keywords:** Thyroid, Levothyroxine, Total thyroidectomy, Hypothyroidism, Ethanol-free liquid Levothyroxine - levotirsol

## Abstract

**Purpose:**

Since the introduction of an ethanol-free liquid levothyroxine formulation, only few studies assessed its therapeutic efficacy. The present study aimed to compare the efficacy of tablet (T-LT4) versus L-EF-LT4 in achieving the patient-specific TSH target in thyroidectomized patients. The primary objective was to assess whether the required LT4 dose differed between formulations in a real-life setting.

**Methods:**

The study was conducted on 275 patients who underwent total thyroidectomy and were treated with either T-LT4 (n = 152) or L-EF-LT4 (n = 123) between 2020 and 2023. Clinical and biochemical data, including LT4 dose, BMI, TSH levels, and potential causes of malabsorption, were collected. The primary endpoint was the pro-kg LT4 dose required to achieve individualized TSH targets. Multivariate regression analysis was used to evaluate predictors of LT4 requirement.

**Results:**

At final follow-up, 227 patients (82.5%) achieved their TSH target and were under stable doses of LT4 with no significant difference in mean LT4 dose between groups (T-LT4: 1.54 ± 0.35 µg/kg/day; L-EF-LT4: 1.60 ± 0.32 µg/kg/day, *p* = 0.160). TSH levels were comparable (*p* = 0.311). Regression analysis showed that age and BMI were inversely correlated with LT4 dose, while a diagnosis of differentiated thyroid cancer was positively correlated with LT4 dose. Formulation type was not a significant predictor.

**Conclusion:**

In patients with post-surgical hypothyroidism, L-EF-LT4 demonstrated equivalent therapeutic efficacy to T-LT4 in terms of dose requirement and TSH control. These findings support the use of L-EF-LT4 as a viable alternative to tablet LT4, particularly in clinical scenarios where flexible timing of ingestion may improve patient compliance.

## Introduction

Hypothyroidism is a highly prevalent clinical condition worldwide. Chronic autoimmune thyroiditis (CAT) accounts for the majority of cases followed by iatrogenic hypothyroidism, following either thyroidectomy or radioiodine treatment [1]. More rare forms encompass congenital and central hypothyroidism. The gold standard therapy for hypothyroidism, independently from its etiology, is represented by lifelong replacement with Levothyroxine (LT4), due to its efficacy, long-term experience of its benefits, favorable side effect profile, ease of administration, long half-life, and low cost [2, 3]. The tablet LT4 (T-LT4) formulation has been used since 1949 and it is the most used worldwide [4]. Several thyroid-unrelated conditions, such as gastrointestinal disorders (i.e., atrophic gastritis, Helicobacter pylori gastritis, celiac disease, and lactose malabsorption/intolerance), bariatric surgery and concomitant ingestion of other drugs and foods/beverages were reported to potentially reduce LT4 absorption [5, 6]. Following this observation, new LT4 formulations were produced, including liquid solutions (the Liquid Ethanol-based LT4 solution Tirosint, L-EB-LT4, and Parabens-based LT4 solution Tifactor) and soft gel capsules. These new LT4 formulations would provide a potential clinical benefit due to more rapid gastrointestinal absorption [7]. Clinical studies suggest that the liquid ethanol-based LT4 formulation (L-EB-LT4) would be able to revert T-LT4 malabsorption caused by certain drugs, bariatric surgery, food or coffee [8–11]. Most data comparing the efficacy of novel versus T-LT4 formulations derive from studies including hypothyroid subjects with CAT [10–12]. A major limitation of these studies stems from the varying degree of thyroid functional reserve in patients with CAT. Post-total thyroidectomy hypothyroidism would represent a better model, since patients have no residual thyroid tissue and require full replacement therapy with LT4. Only few small sample size studies have compared the performance of T-LT4 and novel LT4 formulations in thyroidectomized patients, with some authors suggesting a better performance of L-EB-LT4 or soft gel formulations [13–18]. A new liquid, ethanol free LT4 formulation (Tirosint®SOL, by IBSA, commercialized in Italy under the name of Levotirsol), was approved by the FDA in 2017. Tirosint®SOL is a liquid ethanol-free LT4 solution (L-EF-LT4) in 85% glycerol in single-dose. Pharmacokinetic studies show that this formulation reaches maximum systemic exposure 30 min earlier than T-LT4 and soft gel capsules and has a 30-min shorter lag time [19–21]. Clinical studies regarding the use of this formulation are still limited, and no data is available regarding thyroidectomised patients.

The aim of the present study was to compare the therapeutic dose of LT4 required to reach and maintain the patient-specific TSH target in athyreotic patients receiving either T-LT4 or L-EF-LT4. The two LT4 formulations were compared by taking into account: i) LT4 daily dose per body weight; ii) patients-related factors potentially influencing LT4 requirement

## Material and methods

### Patients and study design

The clinical records of patients undergoing total thyroidectomy at the Unit of General and Minimally Invasive Surgery of ICS Maugeri (Pavia) and followed at the outpatient Endocrinology Unit of the same Institution between January 2020 and January 2023 were retrospectively examined. Inclusion criteria were i) patients who underwent total thyroidectomy for benign or malignant disease; ii) post thyroidectomy-treatment with either tablet formulation of levothyroxine (T-LT4) or liquid ethanol-free formulation of levothyroxine (Levotirsol, L-EF-LT4). Exclusion criteria were: i) age <18 years old; ii) pregnancy during follow up; iii) post-thyroidectomy hypothyroidism treated by any drug other than T-LT4 and L-EF-LT4.

Data regarding the LT4 dose and formulation chosen, age, weight, height, BMI, were collected at baseline. Data regarding possible causes of malabsorption, including drugs (PPI, statins), anamnesis for IBD, celiac disease, lactose intolerance, atrophic body gastritis (ABG), and bariatric surgery were collected. Histological result and TSH values were collected at first post-surgery follow-up (T1) and patients status was classified as below- TSH target, at TSH target or above TSH target according to their TSH targets. Data regarding body weight, TSH levels and LT4 dose and formulation were collected also at the last available follow-up time (T2), expressed in weeks.

All patients signed an informed consent concerning the future use of their clinical-pathological data for research purposes. This study was formally approved by the Istituti Clinici Scientifici Maugeri IRCCS Ethical Committee (Protocol number CE 2418).

#### Institutional management of post-thyroidectomy hypothyroidism

For all thyroidectomised patients LT4 therapy is started the day after thyroidectomy, and doses are empirically established by endocrinologists. Patients are instructed to take LT4 at the same time every day, on an empty stomach, 30’ before breakfast, at least 2 h before calcium supplementation.

TSH and FT4 are determined 6–8 weeks later (time 1, T1) and levothyroxine dose are titrated when the TSH target is not achieved. TSH targets are different depending on several factors mainly including histological diagnosis, age, desire of pregnancy and comorbidities and were chosen according to the latest ATA guidelines on thyroid cancer and hypothyroidism [2, 22, 23]. For the purpose of the present study euthyroidism was defined as achievement of the individualized TSH target (i.e. not requiring any further dose-adjustment) rather than a TSH level within the normal range (0.4–4.0).

#### Statistical analysis

Statistical analysis was performed using the SPSS Software version 29 (SPSS, Inc.). Between-groups comparisons were performed using the Student’s t-test for unpaired data and the Mann–Whitney U-test according to a normal or a non-parametric distribution. Within-group comparisons were performed using the Student’s t-test for paired data and the Wilcoxon’s test according to a normal or a non-parametric distribution. Frequencies among groups were performed using the χ2-test with Fisher’s correction when appropriate. A *p*-value < 0.05 was considered significant. A linear regression model was designed including the per/kg LT4 dose among euthyroid patients as dependent variable and gender, age, BMI, presence of potential causes of malabsorption, histological diagnosis and LT4 formulation as covariates.

Sample size was calculated considering a non-inferiority limit of −0.2 mcg/kg/day of LT4 among euthyroid patients between T-LT4 and L-LT4, a 90% power and a one-sided 95% confidence interval. The required sample size was of at least 78 patients per group [24].

## Results

### Baseline characteristics of patients

According to the inclusion and exclusion criteria, 275 patients were enrolled. As shown in Table 1, 152 patients (55.3%) started treatment with T-LT4, while 123 (44.7%) with L-EF-LT4. At baseline, the two group were homogeneous, and there were no significant differences in terms of sex, age, BMI, histologic diagnosis, and the rate of potential causes of malabsorption. Starting LT4 dose was comparable in pat ients treated with T-LT4 or L-EF-LT4.

**Table 1:** Baseline data of patients treated with tablet-LT4 (T-LT4) or liquid-Ethanol free-LT4 (L-EF-LT4)

	Total	T-LT4	L-EF-LT4	*p* value
N. of patients	275	152 (55.3%)	123 (44.7%)	
Sex F/M, n (%)	219/56 (79.6%/20.4%)	120/32 (78.9%/21.1%)	99/24 (80.5%/19.5%)	0.752
Age (mean ± SD)	53.2 ± 12.9	54.2 ± 12.6	52.1 ± 13.2	0.183
BMI (mean ± SD)	26.0 ± 5.3	26.0 ± 5.3	26.0 ± 5.2	0.983
Starting LT4 dose (µg/Kg/day) (mean ± SD)	1.5 ± 0.2	1.4 ± 0.3	1.5 ± 0.2	0.624
Differentiated Thyroid Cancer, n (%)	78 (28.4%)	42 (27.6%)	36 (29.3%)	0.765
Causes of potential malabsorption, n (%)	76 (27.6%)	43 (28.3%)	33 (26.8%)	0.788

### First post-thyroidectomy follow-up (T1)

**Fig. 1:**
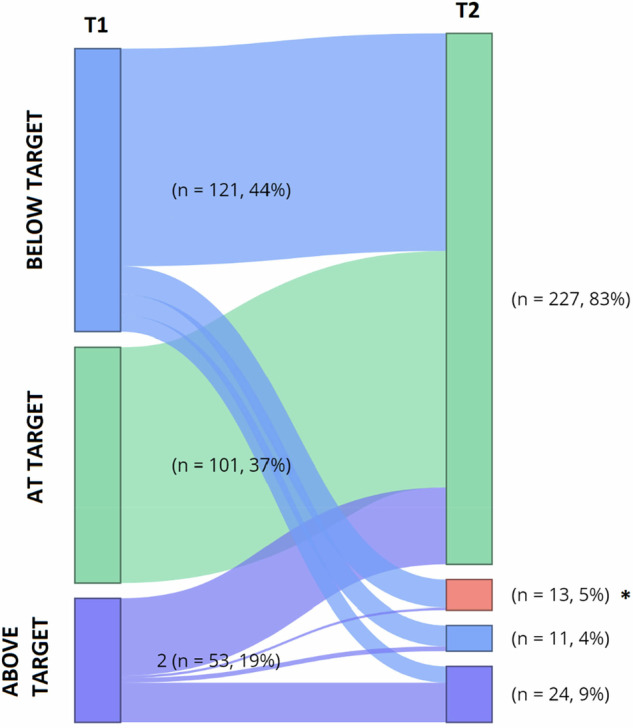
Sankey Diagram representing patient’s status at T1 and T2. * Patients lost at follow-up after T1

As shown in Fig. 1, at T1 118 patients (42.9%) were below TSH target, 104 (37.8%) achieved their TSH target, and 53 (19.3%) were above their TSH target. No significant differences in the rate of euthyroid patients could be observed between the patients taking T-LT4 or L-EF-LT4 (40.8% in the T-LT4 group versus 31.7% in the L-EF-LT4 group, p = 0.120). At T1, LT4 dose adjustments were performed according to TSH target levels.

### Last post-thyroidectomy follow-up (T2)

As shown in Fig. 1, 13 patients dropped-out because of lack of follow-up visit after T1, so data regarding T2 were available for 262 out of 275 patients. At T2, 227 patients had achieved their TSH target (82.5%), 11 (4.0%) were still below their TSH target and 24 (8.7%) were over-treated. Median time of achievement of TSH target was 13 (8–57) weeks. The rate of patients achieving their TSH target at last follow-up was similar between T-LT4 and L-EF-LT4 [131 patients (89.7%) for T-LT4 versus 96 patients (82.8%) for L-EF-LT4, *p* = 1.00)

**Fig. 2:**
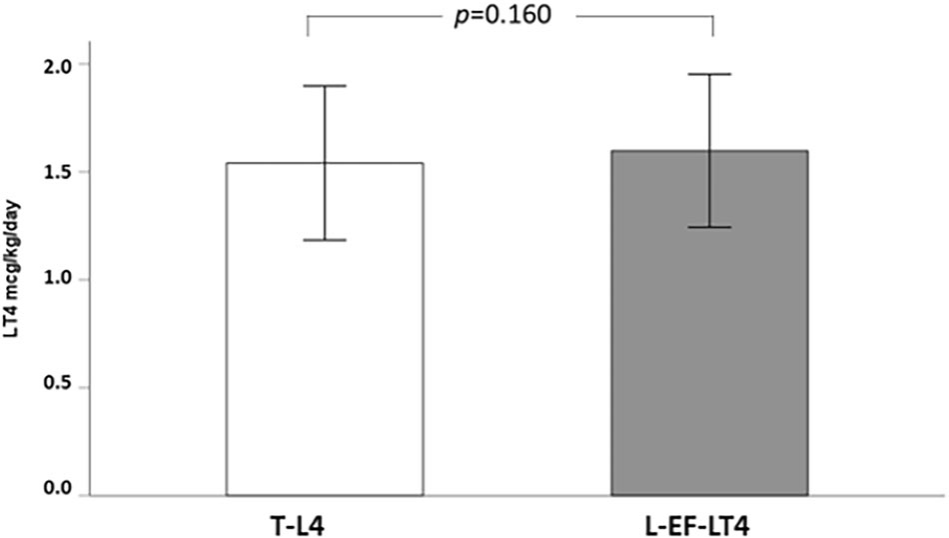
Comparison of mean daily per-kg dose between Tablet Levothyroxine (T-LT4) and Liquid Ethanol-Free Levothyroxine (L-EF-LT4) among patients reaching their TSH target at T2. Bars represent mean values and brackets represent standard Deviation

As shown in Table 2, when considering only the 227 patients who had reached their TSH target at T2, TSH was similar between patients treated with T-LT4 [TSH 1.00 mUI/L (0.56–2.07)] vs L-EF-LT4 [TSH 1.38 mUI/L (0.44–2.40), *p* = 0.311]. As shown in Fig. 2, among the at target subjects the mean LT4 per/kg dose was similar between T-LT4 (1.54 ± 0.35 µg/Kg/day) and L-EF-LT4 (1.60 ± 0.32 µg/Kg/day, p = 0.160).

**Table 2:** Comparison of the clinical and biochemical characteristics of patients reaching their TSH target at last follow-up treated with T-LT4 or L-LT4

	Total	T-LT4	L-EF-LT4	*p* value
N. of patients	227	131 (57.7%)	96 (42.3%)	
Sex F/M n (% of Males)	184/43 (18.9%)	105/26 (19.8%)	79/17 (17.7%)	0.685
Age (mean ± SD)	53.2 ± 12.5	53.6 ± 12.7	52.6 ± 12.2	0.535
BMI (mean ± SD)	26.3 ± 5.4	26.3 ± 5.5	26.3 ± 5.3	0.964
LT4 dose (µg/Kg/day) (mean ± SD)	1.6 ± 0.3	1.5 ± 0.4	1.6 ± 0.3	0.160
TSH (µU/ml), median (25^th^–75^th^ centile)	1.13 (0.51–2.16)	1.00 (0.56–2.07)	1.38 (0.44–2.40)	0.311
Differentiated Thyroid Cancer, n (%)	72 (31.7%)	40 (30.5%)	32 (33.3%)	0.652
Causes of potential malabsorption n (%)	65 (28.6%)	37 (28.2%)	28 (29.2%)	0.879

Among the 12 DTC patients (7 treated with T-LT4 and 5 treated with L-EF-LT4) who required TSH suppression according to ATA guidelines, the required LT4 per/kg dose was similar between the two formulations (1.74 ± 0.27 mcg/kg for T-LT4 and 1.79 ± 0.27 mcg/kg for L-EF-LT4, p = 0.721).

To identify clinical variables potentially associated with LT4 per/kg daily dose among subjects reaching their TSH target, a linear regression model was built with LT4 dose as dependent variable and age, gender, BMI, causes of malabsorption, histological diagnosis and LT4 formulation as independent predictors. As shown in Table 3, age and BMI were significantly and independently correlated with a lower LT4 dose, while a histological diagnosis of DTC was related with a higher LT4 requirement. Gender, presence of possible causes of Malabsorption and LT4 formulations were not significantly related with differences in LT4 requirement.

**Table 3:** Multiple Regression analysis with LT4 daily per/kg dose in subjects reaching their TSH target and age, gender, BMI, causes of Malabsorption, presence of Differentiated Thyroid Cancer (DTC) at histology and LT4 formulation as independent predictors

		95.0% C.I. for β		*p* value
	β	Lower	Upper	
BMI (kg/m^2^)	−0.408	−0.518	−0.298	**<0.001**
Gender (Male vs Female)	0.095	−0.018	0.208	0.098
Age (years)	−0.303	−0.455	−0.189	**<0.001**
Malabsorption (Yes vs No)	−0.014	−0.126	0.098	0.811
Formulation (T-LT4 vs L-EF-LT4)	0.076	−0.032	0.185	0.166
DTC vs Benign Thyroid Disease	0.216	0.105	0.327	**<0.001**

Bold values indicate *p* values lower than 0.05 (considered significant)

## Discussion

The results of the present study show that, in thyroidectomized patients receiving LT4 therapy 30 min before breakfast and achieving their TSH target with similar levels of serum TSH, no significant difference in the LT4 µg/kg/day dose was observed between patients treated with L-EF-LT4 as compared to those treated with T-LT4.

Studies evaluating L-EF-LT4 are currently limited. Two studies performed in healthy volunteers showed that L-EF-LT4 absorption is not impacted by concomitant PPI therapy [25] or by the concomitant consumption of meals [26]. Moreover, a study including a small group of patients with both primary hypothyroidism due to CAT and central hypothyroidism reported improved Quality of Life and a slight increase in FT4 levels following the switch from T-LT4 to L-EF-LT4 [27]. Another study compared a small group of patients with overt hypoithyroidism due to either CAT or total thyroidectomy treated with T-LT4 with a group treated with an ethanol-free T4 formulation in drops (T4® drops), showing an overall therapeutic equivalence [28]. Only one study compared L-EF-LT4 with L-EB-LT4 (Tirosint). The results showed that 48 patients with CAT who were on stable treatment with L-EB-LT4 formulation who switched to L-EF-LT4 at the same dose experienced an increase in serum TSH levels accompanied by a decrease in circulating FT4 levels as compared to those who continued with the L-EB-LT4 formulation [29].

Most of the available literature regarding the comparison between T-LT4 and novel formulations takes into account L-EB-LT4 formulations or Softgel capsules. Most studies focused on specific populations at high risk for LT4 malabsorption, such as patients taking specific drugs [10, 11], patients with gastrointestinal disorders [30–32], patients undergoing bariatric surgery [33, 34], patients taking LT4 in short proximity of meals [12, 17, 35] or children with congenital hypothyroidism [36]. A minority of studies included specifically patients with CAT and no obvious cause of malabsorption, suggesting a better performance of L-EB-LT4 [37]. Moreover, only few studies specifically included patients with post-surgical hypothyroidism [5, 13–17].

In particular, three randomized controlled studies performed in thyroidectomised patients showed a modest, although significant, better performance of L-EB-LT4 when compared with T-LT4 [14, 15, 18].

A 2017 randomized trial showed that thyroidectomised patients receiving L-EB-LT4 had higher scores at several quality of life questionnaires and achieved slightly lower TSH levels when compared with T-LT4 [14]. A randomized controlled trial including 104 patients thyroidectomised for DTC showed that 24 months after surgery patients treated with T-LT4 had a slightly higher TSH level when compared with those treated with L-EB-LT4 [15]. Another randomized trial including 105 thyroidectomized patients without malabsorption randomly allocated to L-EB-LT4 or T-LT4 at the same starting dose, showed that patients on L-EB-LT4 had lower TSH values during follow-up [18].

Moreover, a 2022 study showed that long-term therapeutic adherence, although overall satisfactory, was significantly higher among thyroidectomised patients treated L-EB-LT4 than those treated with T-LT4 [38].

A 2015 study showed an overall non-inferiority of L-EB-LT4 versus T-LT4 formulations among 59 patients thyroidectomised for DTC [13]. Similarly, a longitudinal crossover study, showed that patients who switched from T-LT4 to Soft gel formulations achieved slightly lower TSH values [16].

At present, no study directly compared the performance of the L-EF-LT4 formulation with the T-LT4. At difference with the above cited studies all performed with L-EB-LT4, the results of the present study demonstrate an overall similarity of L-EF-LT4 when compared to T-LT4. Our results could be at least in part explained by the study design. Indeed, we specifically analysed the LT4 pro-kg requirement among patients in full replacement dose reaching their TSH target. This target was individually tailored according to several clinical parameters, including histological diagnosis, age, presence of comorbidities, and desire of pregnancy. The here reported results are clinically relevant since they confirm the safety and efficacy of the novel L-EF-LT4. These data support L-EF-LT4 as an option in the treatment of thyroidectomised patients, in a real-life context. As a further point, preliminary evidence supports the satisfactory pharmacokinetic profile of L-EF-LT4 even when taken in proximity of meals [25, 26]. Thus, it could be hypothesized that similar results in terms of TSH target-reaching could be achieved even in patients that do not wait the conventional 30 min before breakfast for therapy assumption, as typically recommended for T-LT4. Given the retrospective nature of the present study, no direct assessment of patient compliance was available. Nevertheless, recent studies suggest that liquid LT4 formulations could improve patients compliance, especially during long follow-up observations [35, 39].

As a further finding, our results showed that LT4 requirements were inversely proportional to BMI and age, while patients with DTC required higher doses. These results are in line with a great body of evidence showing that elderly subjects and those with a higher BMI require lower pro-kg LT4 doses [5, 40, 41]. On the other hand, presence of evident causes of malabsorption and gender did not impact on LT4 dose.

In conclusion, the results obtained in a large series of patients with post-surgical hypothyroidism receiving a full LT4 replacement dose indicate an overall superimposable therapeutic efficacy of the T-LT4 and the L-EF-LT4 formulations. Future studies with L-EF-LT4 will be required to further evaluate the potential superiority of such a formulation in relation to concomitant/delayed breakfast/meal ingestion.

## Data Availability

Some or all data used during the study are available from the corresponding author by request.

## References

[1] P.N. Taylor, D. Albrecht, A. Scholz, G. Gutierrez-Buey, J.H. Lazarus, C.M. Dayan et al., Global epidemiology of hyperthyroidism and hypothyroidism. Nat. Rev. Endocrinol. **14**(5), 301–316 (2018). 10.1038/nrendo.2018.18. 29569622 10.1038/nrendo.2018.18

[2] J. Jonklaas, A.C. Bianco, A.J. Bauer, K.D. Burman, A.R. Cappola, F.S. Celi et al., Guidelines for the treatment of hypothyroidism: prepared by the american thyroid association task force on thyroid hormone replacement. Thyroid **24**(12), 1670–1751 (2014). 10.1089/thy.2014.0028. 25266247 10.1089/thy.2014.0028 PMC4267409

[3] F. Magri, L. Chiovato, L. Croce, M. Rotondi, Thyroid hormone therapy for subclinical hypothyroidism. Endocrine **66**(1), 27–34 (2019). 10.1007/s12020-019-02039-z. 31617163 10.1007/s12020-019-02039-z

[4] L.H. Duntas, Seven decades of levothyroxine: a comprehensive profile. Adv. Ther. **36**(Suppl 2), 27–29 (2019). 10.1007/s12325-019-01081-7. 31485976 10.1007/s12325-019-01081-7 PMC6822813

[5] P. Miccoli, G. Materazzi, L. Rossi, Levothyroxine therapy in thyrodectomized patients. Front. Endocrinol. (Lausanne) **11**, 626268 (2020). 10.3389/fendo.2020.626268. 33584551 10.3389/fendo.2020.626268 PMC7878675

[6] P. Trimboli, N. Ossola, A. Torre, F. Mongelli, M. Quarenghi, C. Camponovo et al., The performance of levothyroxine tablet is impaired by bariatric surgery. Endocrine **80**(3), 563–569 (2023). 10.1007/s12020-022-03289-0. 36581744 10.1007/s12020-022-03289-0 PMC10199827

[7] C. Virili, P. Trimboli, F. Romanelli, M. Centanni, Liquid and softgel levothyroxine use in clinical practice: state of the art. Endocrine **54**(1), 3–14 (2016). 10.1007/s12020-016-1035-1. 27473098 10.1007/s12020-016-1035-1

[8] C. Virili, L. Giovanella, P. Fallahi, A. Antonelli, M.G. Santaguida, M. Centanni et al., Levothyroxine therapy: changes of TSH Levels by switching patients from tablet to liquid formulation. A systematic review and meta-analysis. Front. Endocrinol. (Lausanne) **9**, 10 (2018). 10.3389/fendo.2018.00010. 29434573 10.3389/fendo.2018.00010 PMC5790785

[9] C. Virili, P. Trimboli, M. Centanni, Novel thyroxine formulations: a further step toward precision medicine. Endocrine **66**(1), 87–94 (2019). 10.1007/s12020-019-02049-x. 31617168 10.1007/s12020-019-02049-x

[10] R. Vita, G. Saraceno, F. Trimarchi, S. Benvenga, Switching levothyroxine from the tablet to the oral solution formulation corrects the impaired absorption of levothyroxine induced by proton-pump inhibitors. J. Clin. Endocrinol. Metab. **99**(12), 4481–4486 (2014). 10.1210/jc.2014-2684. 25259910 10.1210/jc.2014-2684

[11] S. Benvenga, F. Di Bari, R. Vita, Undertreated hypothyroidism due to calcium or iron supplementation corrected by oral liquid levothyroxine. Endocrine **56**(1), 138–145 (2017). 10.1007/s12020-017-1244-2. 28155174 10.1007/s12020-017-1244-2

[12] C. Cappelli, I. Pirola, L. Daffini, A. Formenti, C. Iacobello, A. Cristiano et al., A double-blind placebo-controlled trial of liquid thyroxine ingested at breakfast: results of the TICO study. Thyroid **26**(2), 197–202 (2016). 10.1089/thy.2015.0422. 26586610 10.1089/thy.2015.0422

[13] M. Giusti, L. Mortara, N. Machello, E. Monti, G. Pera, M. Marenzana, Utility of a liquid formulation of levo-thyroxine in differentiated thyroid cancer patients. Drug. Res. (Stuttg.) **65**(6), 332–336 (2015). 10.1055/s-0034-1384535. 25020105 10.1055/s-0034-1384535

[14] C.P. Lombardi, R. Bocale, A. Barini, A. D’Amore, M. Boscherini, R. Bellantone, Comparative study between the effects of replacement therapy with liquid and tablet formulations of levothyroxine on mood states, self-perceived psychological well-being and thyroid hormone profile in recently thyroidectomized patients. Endocrine **55**(1), 51–59 (2017). 10.1007/s12020-016-1003-9. 27388589 10.1007/s12020-016-1003-9

[15] C. Cappelli, I. Pirola, E. Gandossi, C. Casella, D. Lombardi, B. Agosti et al., TSH variability of patients affected by differentiated thyroid cancer treated with levothyroxine liquid solution or tablet form. Int. J. Endocrinol. **2017**, 7053959 (2017). 10.1155/2017/7053959. 28572820 10.1155/2017/7053959 PMC5441121

[16] V. Di Donna, R.M. Paragliola, C. de Waure, G. Papi, A. Pontecorvi, S.M. Corsello, Is levothyroxine requirement the same for tablet and soft gel formulations?. Endocrine **59**(2), 458–460 (2018). 10.1007/s12020-017-1311-8. 28466401 10.1007/s12020-017-1311-8

[17] C. Cappelli, I. Pirola, E. Gandossi, A. Cristiano, L. Daffini, B. Agosti et al., Thyroid hormone profile in patients ingesting soft gel capsule or liquid levothyroxine formulations with breakfast. Int. J. Endocrinol. **2016**, 9043450 (2016). 10.1155/2016/9043450. 27313613 10.1155/2016/9043450 PMC4904100

[18] P. Fallahi, S.M. Ferrari, G. Materazzi, F. Ragusa, I. Ruffilli, A. Patrizio et al., Oral L-thyroxine liquid versus tablet in patients submitted to total thyroidectomy for thyroid cancer (without malabsorption): a prospective study. Laryngoscope Investig. Otolaryngol. **3**(5), 405–408 (2018). 10.1002/lio2.186. 30410995 10.1002/lio2.186 PMC6209618

[19] P. Colucci, P. D’Angelo, G. Mautone, C. Scarsi, M.P. Ducharme, Pharmacokinetic equivalence of a levothyroxine sodium soft capsule manufactured using the new food and drug administration potency guidelines in healthy volunteers under fasting conditions. Ther. Drug. Monit. **33**(3), 355–361 (2011). 10.1097/FTD.0b013e318217b69f. 21516059 10.1097/FTD.0b013e318217b69f

[20] M. Tanguay, J. Girard, C. Scarsi, G. Mautone, R. Larouche, Pharmacokinetics and comparative bioavailability of a levothyroxine sodium oral solution and soft capsule. Clin. Pharmacol. Drug. Dev. **8**(4), 521–528 (2019). 10.1002/cpdd.608. 30153382 10.1002/cpdd.608 PMC6585626

[21] D. Al-Numani, C. Scarsi, M.P. Ducharme, Levothyroxine soft capsules demonstrate bioequivalent pharmacokinetic exposure with the European reference tablets in healthy volunteers under fasting conditions. Int. J. Clin. Pharmacol. Ther. **54**(2), 135–143 (2016). 10.5414/CP202485. 26754305 10.5414/CP202485

[22] B.R. Haugen, E.K. Alexander, K.C. Bible, G.M. Doherty, S.J. Mandel, Y.E. Nikiforov et al., 2015 American Thyroid Association Management guidelines for adult patients with thyroid nodules and differentiated thyroid cancer: the american thyroid association guidelines task force on thyroid nodules and differentiated thyroid cancer. Thyroid **26**(1), 1–133 (2016). 10.1089/thy.2015.0020. 26462967 10.1089/thy.2015.0020 PMC4739132

[23] J.R. Garber, R.H. Cobin, H. Gharib, J.V. Hennessey, I. Klein, J.I. Mechanick et al., Clinical practice guidelines for hypothyroidism in adults: cosponsored by the American Association of Clinical Endocrinologists and the American Thyroid Association. Endocr. Pract. **18**(6), 988–1028 (2012). 10.4158/EP12280.GL. 23246686 10.4158/EP12280.GL

[24] S.A. Julious, Sample sizes for clinical trials with normal data. Stat. Med. **23**(12), 1921–1986 (2004). 10.1002/sim.1783. 15195324 10.1002/sim.1783

[25] C. Seng Yue, C. Scarsi, E. Bettazzi, G. Mautone, F.S. Celi, M. Ducharme, Proton pump inhibitors do not affect the bioavailability of a novel liquid formulation of levothyroxine. Endocr. Pract. **30**(6), 513–520 (2024). 10.1016/j.eprac.2024.03.388. 38554774 10.1016/j.eprac.2024.03.388

[26] M. Ducharme, C. Scarsi, E. Bettazzi, G. Mautone, Y. Lewis, F.S. Celi, A novel levothyroxine solution results in similar bioavailability whether taken 30 or just 15 min before a high-fat high-calorie meal. Thyroid **32**(8), 897–904 (2022). 10.1089/thy.2021.0604. 35469428 10.1089/thy.2021.0604 PMC9419984

[27] K. Bornikowska, M. Gietka-Czernel, D. Raczkiewicz, P. Glinicki, W. Zgliczyński, Improvements in quality of life and thyroid parameters in hypothyroid patients on ethanol-free formula of liquid levothyroxine therapy in comparison to tablet LT4 form: an observational study. J. Clin. Med. **10**(22), 5233 (2021). 10.3390/jcm10225233. 34830515 10.3390/jcm10225233 PMC8624226

[28] G.K. Markantes, K. Dimitropoulos, I. Mamali, I. Tseti, G. Sakellaropoulos, K.B. Markou et al., Therapeutic equivalence of a new preparation of liquid levothyroxine with tablets in patients with overt primary hypothyroidism. Eur. Thyroid. J. **10**(1), 59–64 (2021). 10.1159/000508216. 33777820 10.1159/000508216 PMC7983587

[29] E. Gatta, V. Maltese, I. Pirola, E. Gandossi, I. Silvestrini, M. Ugoccioni et al., Are liquid levothyroxine formulations comparable? The LETI study. Thyroid. Res. **18**(1), 17 (2025). 10.1186/s13044-025-00236-9. 40269940 10.1186/s13044-025-00236-9 PMC12020242

[30] S.M. Ferrari, A. Patrizio, V. Mazzi, F. Ragusa, C. Botrini, G. Elia et al., Lactose intolerance and levothyroxine malabsorption: a review of the literature and report of a series of patients treated with liquid L-T4 without lactose. Front. Endocrinol. (Lausanne) **15**, 1386510 (2024). 10.3389/fendo.2024.1386510. 38665263 10.3389/fendo.2024.1386510 PMC11044000

[31] P. Fallahi, S.M. Ferrari, G. Elia, F. Ragusa, S.R. Paparo, A. Antonelli, L-T4 therapy in enteric malabsorptive disorders. Front. Endocrinol. (Lausanne) **12**, 626371 (2021). 10.3389/fendo.2021.626371. 33708175 10.3389/fendo.2021.626371 PMC7940821

[32] M. Castellana, C. Castellana, L. Giovanella, P. Trimboli, Prevalence of gastrointestinal disorders having an impact on tablet levothyroxine absorption: should this formulation still be considered as the first-line therapy?. Endocrine **67**(2), 281–290 (2020). 10.1007/s12020-019-02185-4. 31953721 10.1007/s12020-019-02185-4

[33] M. Almukainzi, R. AlQahtani, R. Alanazi, R. Alamri, H. Alayed, Insight of the biopharmaceutical implication of sleeve gastrectomy on levothyroxine absorption in hypothyroidism patients. Obes. Surg. **34**(1), 192–197 (2024). 10.1007/s11695-023-06970-z. 38091193 10.1007/s11695-023-06970-z

[34] P. Fallahi, S.M. Ferrari, S. Camastra, U. Politti, I. Ruffilli, R. Vita et al., TSH normalization in bariatric surgery patients after the switch from L-Thyroxine in tablet to an oral liquid formulation. Obes. Surg. **27**(1), 78–82 (2017). 10.1007/s11695-016-2247-4. 27272506 10.1007/s11695-016-2247-4

[35] V. Oteri, S. Volpe, M. Lopes, G. Sceusa, A. Tumminia, A. Belfiore et al., Therapeutic efficacy and patient compliance of levothyroxine liquid and softgel formulations taken with meals: a systematic review. Endocrine **87**(1), 48–58 (2025). 10.1007/s12020-024-04016-7. 39215906 10.1007/s12020-024-04016-7 PMC11739177

[36] R. Ortolano, E. Cantarelli, F. Baronio, V. Assirelli, E. Candela, C. Mastrangelo et al., Comparison between liquid and tablet formulations in the treatment of congenital hypothyroidism up to 3 years of age: the first Italian study. Children (Basel) **11**(9), 1136 (2024). 10.3390/children11091136. 39334669 10.3390/children11091136 PMC11430788

[37] P. Fallahi, S.M. Ferrari, A. Antonelli, In patients with subclinical hypothyroidism while in therapy with tablet L-T4, the liquid L-T4 formulation is more effective in restoring euthyroidism. Endocr. Pract. **23**(2), 170–174 (2017). 10.4158/EP161545.OR. 27849377 10.4158/EP161545.OR

[38] R. Bocale, G. Desideri, A. Barini, A. D’Amore, M. Boscherini, S. Necozione et al., Long-term adherence to levothyroxine replacement therapy in thyroidectomized patients. J. Clin. Med. **11**(15), 4296 (2022). 10.3390/jcm11154296. 35893387 10.3390/jcm11154296 PMC9332058

[39] C. Cappelli, R. Castello, F. Marini, A. Paoletta, M. Marchetti, M. Saullo et al., Adherence to levothyroxine treatment among patients with hypothyroidism: a Northeastern Italian Survey. Front. Endocrinol. (Lausanne) **9**, 699 (2018). 10.3389/fendo.2018.00699. 30532737 10.3389/fendo.2018.00699 PMC6265311

[40] F. Santini, A. Pinchera, A. Marsili, G. Ceccarini, M.G. Castagna, R. Valeriano et al., Lean body mass is a major determinant of levothyroxine dosage in the treatment of thyroid diseases. J. Clin. Endocrinol. Metab. **90**(1), 124–127 (2005). 10.1210/jc.2004-1306. 15483074 10.1210/jc.2004-1306

[41] J. Jonklaas, Sex and age differences in levothyroxine dosage requirement. Endocr. Pract. **16**(1), 71–79 (2010). 10.4158/EP09257.OR. 19833578 10.4158/EP09257.OR

